# Communication in production animal medicine: modelling a complex interaction with the example of dairy herd health medicine

**DOI:** 10.1186/2046-0481-64-8

**Published:** 2011-07-20

**Authors:** Joachim L Kleen, Owen Atkinson, Jos PTM Noordhuizen

**Affiliations:** 1 CowConsult, Hochfeldhof, D-26670 Uplengen, Germany; 2 Lambert, Leonard and May Farm Vets, Whitchurch, SY13 4AQ UK; 3 VACQA-International, Santarém, Portugal; associated with the Population Medicine Group, Lyon National Veterinary School, Marcy l'Etoile, France; 4 Veterinary & Animal Sciences department of the Charles Stuart University, Wagga Wagga, Australia

## Abstract

**Background:**

The importance of communication skills in veterinary medicine is increasingly recognised. Appropriate communication skills towards the client are of utmost importance in both companion animal practice and production animal field and consultancy work. The need for building a relationship with the client, alongside developing a structure for the consultation is widely recognised and applies to both types of veterinary practice.

**Results:**

Veterinary advisory practice in production animal medicine is, however, characterised by a more complex communication on different levels. While the person-orientated communication is a permanent process between veterinarian and client with a rather personal perspective and defines the roles of interaction, the problem-orientated communication deals with emerging difficulties; the objective is to solve an acute health problem. The solution - orientated communication is a form of communication in which both veterinarian and client address longstanding situations or problems with the objective to improve herd health and subsequently productivity performance. All three forms of communication overlap.

**Conclusions:**

Based on this model, it appears useful for a veterinary practice to offer both a curative and an advisory service, but to keep these two separated when deemed appropriate. In veterinary education, the strategies and techniques necessary for solution orientated communication should be included in the teaching of communication skills.

## Background

### Modelling the communication between client and veterinarian

In both human and veterinary medicine, communication skills have received growing attention over the recent years [1]. Over the past 20 years, the importance of communication skills (CS) in human medicine has increasingly been recognized [2]. Various reports describe the importance for Good Medical Practice. It has been shown that CS are relevant for patients' perception of satisfaction [3] and patients' compliance to treatment protocols [4]. The role of communicating health risks, rationale of a therapy and long term-strategy is deemed important and reported for different diseases such as stroke [5] or osteoporosis [6].

In veterinary medicine, various reports and papers have highlighted the relevance of communication skills in the context of veterinary education, clinical science and practice management [7-10]. Professional communication skills refer to the ability of the veterinarian to communicate appropriately and effectively with clients [11]. It has been described as a core clinical skill, as it will not only influence the client-veterinarian relationship, but also directly influence the success of the consultation and the following therapy or other intervention [1,12]. Problems in interpersonal communication will affect the client-veterinarian relationship negatively [13].

A widely recognised model describing the interaction in a medical environment is the Calgary-Cambridge model, which was originally developed to describe and define the necessary communication skills in human medicine [12]. It is used in a large number of veterinary schools and colleges to demonstrate the appropriate communication between veterinarian and client [8]. The model focuses on the consultation as such and describes it as a process with a helical character, as veterinarian and client interact with each other. While the consultancy itself has to be structured appropriately, the model also describes the need for both client and veterinarian to build up a working relationship. The Calgary-Cambridge model was originally designed for teaching communication skills to students; other models published in connection with the consultation process focus on practical aspects, like decision making [14].

The Calgary-Cambridge model of the consultation process applies to companion animal medicine, as well as to most aspects of "traditional", curative, farm animal practice. Emergencies, acutely sick farm animals and some management related issues involve similar steps as follows: the opening of the consultation, gathering information, followed by examination, planning and execution. All these aspects involve both the veterinarian and the client. The need to observe the structure of the consultation as well as the relationship with the client is therefore valid in both environments. In companion animal medicine, the driving force for client-veterinarian engagement is the health of the individual animal, implying the life quality of the owner [13,15]. The aim of farm animal practice is, however, different. Economic decisions are of higher relevance and improvement of health status and consequently raising the productivity of a production animal unit is the goal of veterinary medicine in this respect. While modelling the consultation is certainly helpful, in veterinary advisory practice the communication between veterinarian and owner goes beyond that: long term strategies are common whenever it comes to production animals while knowledge exchange and education of farmers are important. The question of what instruments the veterinarian in veterinary advisory practice may use and how communication should best be organized is therefore to be examined in this paper. It will give a review on the current literature and, based on a model to illustrate the communication processes, draw conclusions on practice organization and veterinary education.

### Role of communication in veterinary advisory practice on dairy farms

The veterinarian, who is involved in farm (production) animal medicine, is often faced with problems going beyond one defined question and the area of purely clinical activities [16]. Production animal medicine is focussed on a decrease of disease incidence and prevalence, thereby reducing losses and production costs and increasing animal health and subsequently farm income. In other words, production animal medicine is oriented towards increasing the proportion of healthy animals in a herd by different veterinary activities like monitoring and early diagnostic warning, problem analysis, intervention and prevention [17]. Among others, it involves monitoring of feeding regimes and advice on ration composition, analysis of fertility data and management, counselling on milk-quality and milking technique or consultancy on planning, constructing and organizing buildings on the farm. By these long-term activities, production animal medicine is increasingly turning into veterinary advisory practice, focussing on the well-being and performance of the herd instead of the health status of the individual animal. This integrated approach to herd health and productivity has a lot of names: it has been termed Herd Health Planning [18], Herd Health and Production Management [17], Veterinary Herd Health Management [16] or programmes of Veterinary Quality Risk Management [19]. Veterinary advisory practice is focusing on risks in order to prevent damage instead of trying to manage damage once it has occurred. The basis for success in this veterinary advisory practice is undeniably the knowledge and skills of the practicing veterinarian in (clinical) veterinary matters and zootechnics, i.e. the knowledge of feeding, genetics, farm management and other related areas [16,20,21]. In a dairy herd, there are various disorders prevalent at the same time, e.g. reproductive disorders, mastitis, claw lesions, digestive or metabolic disorders. In this complex environment of production animal practice, the veterinarian needs to take these issues into account, but also must be able to present them to the client in a way the client cannot ignore [22].

Consequently, the motivation for engagement in production animal medicine, such as on dairy farms, is also different from that found in companion animal medicine. Valeeva and others [23], for example, report on a joint motivation to improve udder health: next to the internal, non-monetary factors directly related to animal health, economic factors are important. Here, pending losses are generally more motivating than possible gains. Whereas financial concerns are to a certain extent important in companion animal medicine, they play a key role in the production animal sector. For example, losses due to clinical mastitis are variable and may range from € 17 to € 198 per cow per year, as reviewed by Hogeveen and others [24] in the example of Dutch dairy farms. In the latter case, this would amount to losses of € 0, 02 per kg of milk produced. Moreover, animal welfare considerations are important for animal production: not only do the internal factors on the farm have to be considered, but also the increasing concern of the public regarding the provenance of food of animal origin [16,25].

Adding to the complexity of communication on dairy farms are the different styles of farming, e.g. pasture based systems versus highly intensive feedlot systems or family farms versus large dairy operations held by companies. It is therefore becoming increasingly clear that different styles of communication are required to reach different client individuals [21,26]. It has therefore to be concluded that every model depicting the communication process in veterinary advisory practice has to account for differences between farming styles and the farmers' personalities. Based on 24 interviews, Jansen and others [27] distinguished four types of attitudes among dairy farmers, depending on attitude towards outside information and inclination to adapt changes in behaviour. The group distinguished (1) reclusive traditionalists, (2) pro-activists, (3) wait-and-see-ers and (4) do-it-yourself-ers.

The above mentioned veterinary advisory practice therefore goes beyond the single consultation towards a more complex process involving the veterinarian, the farmer as client and, increasingly, specialists from adjacent fields, hence forming advisory teams [19,28]. Veterinarians have to adopt techniques to facilitate a systematic management approach, integrating the aforementioned factors [20,29].

The veterinarian engaged in veterinary advisory practice does not only need to have acquired certain professional skills like correct interpretation of production data, feed ration calculations and assessment of herd health status, but is also involved in a different kind of communication process with his client. First, the interpersonal communication between veterinarian and farmer involves personal relationship, discussion of emerging problems related to the herd and long-term strategies to improve herd health and performance. Secondly, other methods apart from conversation may be used to broaden the client's knowledge and provide farmers' education in areas concerning the herd health. Jansen and others [30] describe what influence farmers' perception of what is "normal" has on somatic cell count (SCC) in dairy herds. By this phenomenon of "anchoring" a farmer may perceive a critical situation with, e.g. high SCC, as being normal, as he is biased by long-term experience [19]. A number of instruments such as brochures and written standard operating procedures, tailored to the needs and conditions as found on the individual farm, have been described in order to change this perception and shift the framework of what is perceived as being normal [19,25,29,31]. The use of other media for farmer education, such as internet-based education, study groups or workshops has been described by Chase and others [28].

In this context, Jansen and others [32] describe two different strategies to approach farmers. In a central approach, facts are presented on the basis of facts and arguments, thus appeal to understanding and, finally, conviction. A peripheral approach would focus on persuasion techniques and authority of vets and institutions. The authors found both strategies effective but stress the necessity to combine both in order to reach different personalities of the group.

**Figure 1:**
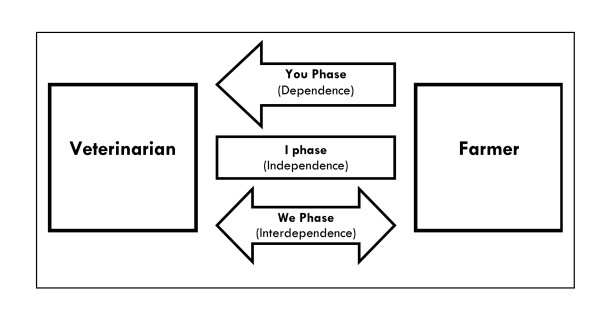
**Levels of interaction between veterinarian and farmer, adapted from Meens (2006)**.

Three different phases of client-veterinarian interaction have been described by Meens ([33] Figure 1). In the "You-Phase", the farmer is heavily depending on the veterinarian and may trust the veterinarian to make decisions and define objectives and actions to be taken. The "I-Phase" is characterised by a farmer acting largely autonomously, with the veterinarian being regarded as provider of certain means or products. The "We-Phase", finally, finds both farmer and veterinarian collaborating to achieve common goals previously defined together. Whether these phases will be active is determined by character of both farmer and vet, the purpose of actual collaboration and the extent to which the collaboration is sought from either party. The phases are independent from each other and may change in the course of collaboration.

To illustrate this model with examples, the "You-Phase" relationship may be similar to that which we might imagine the fictional James Herriott character enjoyed: a farmer waiting expectantly for the vet's diagnosis and advice, and furnishing him with soap, towel and warm water and a cup of tea at the end of the visit. Conversely, the vet in an "I-Phase" relationship is a mere technician. We can imagine the farmer requesting the visit to do a specific job, whether to scan 100 cows for pregnancy or to calve a cow, or write a prescription for medicines, in much the same way that he will place his order for the next load of feed. The vet's opinion is not even sought, much less valued. However, we might imagine a vet in a "We-Phase" relationship as sitting down with the farmer, or the farm team, and engaging in a process where the opinion of all parties is both sought and valued.

In conclusion, dairy herd health management consultancy refers to a complex communication dealing with different motivations, personalities, styles and factors to consider. The consulting production animal veterinarian is delivering more than a technical service; he/she is also functioning as economic advisor, provider of means for farm management and process coach in health and production related issues. Success of the production animal veterinarian is largely dependent on his or her ability to present these complex considerations and clarify them to the client [22]. This kind of advisory practice therefore requires a specific approach towards communication, going beyond the pure consultation as it is found in companion animal medicine. It appears to be useful to integrate this into a model, picturing the relationship between veterinarian and client and explaining the interaction during the advisory process. This model would help to draw conclusions about how a successful veterinary advisory practice should be set up.

### Modelling Communication in Production Animal Medicine

**Figure 2:**
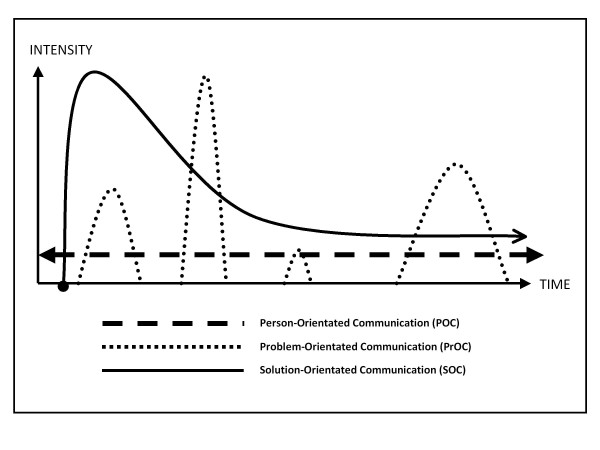
**Time characterization of the different communication types within veterinary advisory practice**.

The interaction of the described environment can be modelled by differentiating the communication into three levels: (1) the person-orientated communication, (2) the problem-orientated communication, and (3) the solution-orientated communication level (Table 1, Figure 2).

**Table 1:** Overview of the three communication types in veterinary advisory practice

Type of communication	Questions adressed	Objective	Time-character	Example
**Person- orientated communication (POC)**	*Who are you?* *How are you?* *What role do you want to take?* *What role do you want me to take?*	Development of personal relationship; building up mutual trust and understanding	Permanent	General product quality issues; Market situation; Discussions of general needs and wishes; Personal matters

**Problem- orientated communication (PrOC)**	*What is your problem?* *What do you want me to do?*	Acute problem that needs attention and resolving	Momentary, transitory	Analysis and solution of a herd fertility problem. Application of "Blitz-Therapy"

**Solution- orientated communication (SOC)**	*What are our goals?* *How can we improve your performance?*	Addressing longstanding problems; improving performance	Long-term	Installing a herd health scheme or a HACCP-like quality risk management approach. Constant monitoring of SCC with an intervention level

#### (1) The person-orientated communication (POC)

The communication orientated on the individual person is the interpersonal communication between veterinarian and his client. This communication is focused on the individual person and will, if successful, be the foundation for mutual understanding and respect. Therefore, the POC defines the respective roles of the participants. Questions to be answered in the context of POC are "*Who are you?" "How are you?" "What role do you want to take?" "What role do you want me to take?"*. Apart from any problem that may, initially, have been the reason for this conversation, this part focuses on the personalities and their responsibilities, and on the collaboration given a specific situation. In particular, newly graduated colleagues with little experience and knowledge regarding the clients have to decipher what function is expected from them by the individual client. If successful, the POC serves as the basis for trust in the interpersonal and, subsequently, professional relationship.

*Example*. By definition, the interpersonal communication is a permanent and unavoidable process. By encoding thoughts into messages and, in turn, receiving and decoding messages, the POC can best be described as a "Frisbee-style" [34] where action and reaction follow each other. This may happen in a conversation about the result of a series of pregnancy diagnoses (PD) during a farm visit:

Veterinarian: "You don't seem to be very happy with the PDs today."

Farmer: "I am not happy with this 50% result."

Veterinarian: "Do you have a possible reason for this in mind?"

Farmer: I was rather busy recently..."

The POC therefore facilitates the collaboration of the persons involved: It will be determined what stage of collaboration is active and what roles are to be taken by vet and farmer (Here: You-level with the farmer depending on the vet). Only a clear understanding in the POC will enable both partners to go forward to the PrOC.

#### (2) The problem-orientated communication (PrOC)

Problem orientated communication between veterinarian and client is communication answering to the questions "*What is your problem?" *and "*What do you want me to do?*" The PrOC is a process that is transitory in character and focuses on emerging and acute problems of the client with the objective to relieve the immediate pressure. The problems can be individual cases (e.g. acute respiratory disease) or cases involving the herd, e.g. an emerging herd mastitis problem. The PrOC very much resembles the communication found in companion animal medicine, as it is described in the Calgary-Cambridge model [12]. The communication process will be closed after a therapy or intervention is planned and, where appropriate, applied. As the PrOC in incited by an emerging problem of which the farmer feels immediate resolution is necessary, the objective of planning the therapy will rather focus on the immediate difficulty and its solution as desired by the client instead of investigating and managing an underlying problem. The PrOC will start rapidly, be very intensive for a comparatively short period of time, and decreases in intensity before being ceased once the problem is solved or restricted and controlled.

*Example*. The conversation regarding poor herd fertility might proceed like this:

Veterinarian: "Do you have a possible reason for this in mind?"

Farmer: "I was rather busy recently, and I didn't have the time to check for heats properly."

Veterinarian: "What did you notice regarding AI-intervals?"

Farmer: "They were rather erratic, a lot of cows coming in heat after 10 or 30 days."

Veterinarian: "It seems the heat detection was not ideal. If we want to take pressure off your time for heat detection, we might consider a timed insemination programme then."

Although this conversation might go on for some time, it is clear that the success of the PrOC depends on the definition of objectives and a particular success by the persons involved in the process. A functioning interpersonal communication is therefore the basis of a successful PrOC.

In the case of individual animals which are acutely diseased, the success will probably be defined by the objective of preserving life, health and productivity of the animal. In the case of herd problems, however, there may be different definitions of what success is. Therefore, the aim of any intervention has to be made clear and agreed upon from both sides; otherwise this may lead to frictions in further collaboration. Here, an example could be *Streptococcus agalactiae *mastitis in a dairy herd that leads to high bulk tank somatic cell counts, consequently to penalties and impairing the economic success of the producer. While the farmer's main objective might be to bring the somatic cell count (SCC) down to a level not interfering with quality standards from the dairy, the veterinarian will necessarily define "success" as complete eradication of the pathogen from the herd, e.g. by means of a "blitz-therapy". This discrepancy in definition of success can lead to frictions once the farmer's objective is reached and the veterinarian pressures to continue milk sampling, for example cows calving in the period after the problem decreased. As said above, the PrOC focuses on the resolution of a problem as perceived by the farmer: It is the veterinarian's responsibility to explain the need for a sustainable solution that not only alleviates the momentary situation but provides a stable herd health situation. Ideally, this will lead to a plan of, in this example, improvement of udder health, hence the start of solution-orientated communication (SOC).

#### (3) The solution-orientated communication (SOC)

In the context of veterinary advisory practice, the communication between veterinarian and farmer may shift away from acute problems that need immediate solving, towards long-term risk management decisions with the purpose of prevention and quality management. The questions that need to be answered in this process are "*What are our goals?" *and "*How can we improve your performance?" *The questions are asked using the pronoun "we", indicating that both veterinarian and farmer are working closely together. This corresponds to the "We-phase", as defined by Meens [33] in which both parties are depending upon each other. Instead of focusing on problems that have already arisen, like in the PrOC, the communication in this phase is directed towards solutions, individually developed for each farm and each situation, and aiming to maintain and improve the level of production, animal health, welfare and quality of the product. We may therefore speak of a solution-orientated communication (SOC).

By character, SOC is a longer-term process. It requires a thorough and conscious preparation as goals and, moreover, the methods for successful collaboration have to be agreed upon. This process of defining the status quo and common goals is a laborious and time-intensive one that requires skills in analysing data and production history. A useful tool in this process can be the assessment of strengths, weaknesses, opportunities and threats (SWOT) [19]. This will lead to a list of issues that need particular attention. Prioritizing these needs warrants careful attention, and highest priority should be attributed to issues that really do matter and that will have an impact on the production quality. Ideally, a successful SOC is based on a stable POC. Mutual trust and understanding are pivotal in establishing the long term process of SOC. Moreover, previous experiences in PrOC with establishing common goals and methods will help in establishing the SOC properly.

*Example*. Once the pressure of rising SCC due to *Streptococcus agalactiae *infections is relieved, a plan might be developed. Goals to be defined could be *(*1) to establish a SCC intervention level (e.g. the bulk milk SCC may increase to up to 170.000 cells/ml before certain measures are taken, and (2) to prevent re-introduction of *Streptococcus agalactiae*. The SOC will then lead to a SWOT, which may define the buying in of cows as a risk. In a third step, measures against this risk are considered, defined and agreed upon, e.g. the compulsory testing of newly bought cows for the pathogen and only buying in from trusted sources. During the SOC, standard operating procedures will be defined and the cooperation between vet and farmer closely planned and defined.

The SOC will require intense labour and preparation in order to get the consultancy process running. After that, the process consists of monitoring status quo and deciding on interventions whenever deemed necessary.

## Discussion and Conclusions

As it has been shown, the three levels of communication in veterinary advisory practice are overlapping and one forms the basis of the other. As the overview (Table 1) shows, these levels cover different aspects of daily veterinary practice. The veterinarian in the production animal sector needs communication skills that are considerably different from those in companion animal medicine. All three levels of communication, the person-orientated, the problem-orientated and the solution-orientated interact with each other and one is the pre-cursor for the other. All together, they form the basis for a successful veterinary advisory practice. Professional communication skills have therefore to be understood as a clinical skill [8]. Only in some countries are communication skills a part of the veterinary curriculum. They are, however, of utmost importance for successful veterinary work and therefore explicitly listed as part of the day-one-skills a new veterinary graduate should have, as defined by the European Association of Establishments for Veterinary Education [35]. The field of communication skills should therefore be made a compulsory part of the education in all veterinary schools. As the CS required in veterinary advisory practice are more complex and long-term in nature than in companion animal practice, this should include not only the PrOC of a consultation, but also the SOC, as it is needed in veterinary advisory production animal practice. It is useful to teach CS with the clear objective of preparing for consultancy work, including skills like writing standard operating procedures and using media for education.

A lack of communication skills may be one of the causes of farmers to drop out from herd fertility schemes, mastitis control programs or herd health advisory programmes, simply because the veterinarians have not been able to communicate their advice in a professional way, or understood the necessary processes [32].

It is probable that veterinary advisory practice is most effective in the framework of an existing working relationship between veterinarian and farmer. However, although the levels of communication do interact with each other, they are basically different levels of collaboration. It might therefore be useful to separate them in daily practice to a certain extent. As the solution-oriented communication deals with different scenarios from those in the purely problem-oriented one, it serves the purpose of herd health programmes to divide these services from each other; for example, there should be no claw trimming during visits. Planning sessions will instead focus on the most important (management) aspect(s) at that particular point of time. This strategy will make clear to both client and veterinarian that these entities are indeed separate services - this may aid in the billing of consultancy work. It appears useful to establish advisory-only vets wherever possible, complementing colleagues in clinical work [29]. Once the veterinarian is able to implement the different types of communication in a proper way, he/she will encounter more success in daily practice and less stress in communicating with the farmer, while the latter may more easily adopt the proposed management changes, as has comparably been established in the U.S. for human medicine [8].

## Competing interests

The authors declare that they have no competing interests.

## Authors' contributions

JK conducted the literature review and developed the model. OA provided expertise on the role of the vet in knowledge transfer. JN provided expertise in conducting veterinary advisory practice. All authors read and approved the final manuscript.
